# Eight years, one patient

**DOI:** 10.1093/oncolo/oyag024

**Published:** 2026-02-03

**Authors:** Tadashi Nishimura, Yoshinobu Matsuda

**Affiliations:** Department of Pulmonary Medicine, NHO Mie Chuo Medical Center, 2158-5 Hisaimyojin-Cho, Tsu City, Mie 514-1101, Japan; Department of Internal Medicine, NHO Kinki Chuo Chest Medical Center, 1180 Kitaku Nagasone-Cho, Sakai City, Osaka 591-8555, Japan

T.N.: In Japan, there is a term known as “*Mund Therapie*,” which uniquely combines the German word for “mouth” and the English word “therapy.” This term refers to the physician’s verbal efforts to persuade patients to accept treatment. However, since the late 1990s, following an increase in medical lawsuits, the term has gradually fallen out of favor and has been replaced by “Informed Consent.” During my residency, conferences were conducted by physicians who followed the concept of “*Mund Therapie*,” which limited patients’ opportunities to participate in decision-making.

I was born in Japan in 1989 and have worked as a pulmonologist in a community hospital for nearly a decade. During this time, the dynamics of the physician-patient relationship have undergone significant transformation, with an increasing emphasis on the importance of shared decision-making. This evolution has paralleled dramatic advancements in the treatment of EGFR-mutated lung cancer.

Among the many changes I have witnessed, one particular event over the past 8 years remains especially memorable: a patient who profoundly shaped an encounter with a patient who profoundly shaped my professional perspective.

Ms. N was 48 years old when she first visited our hospital in 2017. A chest X-ray revealed significant pleural effusion in her left chest, prompting her referral to our facility. I faced the daunting task of delivering a grave diagnosis: “*The cytology of your pleural effusion revealed lung cancer*.”

The image of her reaction and her subsequent words ­continue to resonate with me: “*Please let me hold my grandchild*.”

In Japan, pulmonologists are responsible for treating lung cancer. They not only assume the role of oncologists but also provide palliative care in end-of-life scenarios. As a pulmonology resident, this responsibility weighed heavily on me.

Fortunately, Ms. N was diagnosed with EGFR-mutated lung cancer, providing me with my first experience in managing this specific condition.

At that time, three EGFR tyrosine kinase inhibitors (TKIs) were available in Japan: gefitinib, erlotinib, and afatinib. The guidelines did not specify a first-line treatment, leaving me uncertain and inexperienced in developing an appropriate treatment plan for her. As was customary, we deliberated on the treatment strategy at a conference and ultimately decided to administer afatinib based on evidence of slightly prolonged progression-free survival from the Lux-Lung7 trial.^1^ In that moment, the decision was made by us, not by the patient.

One month into treatment, Ms. N expressed concern over a facial rash attributed to afatinib and requested discontinuation of the drug. Instead, she inquired about a cancer vaccine, despite its limited evidence base. At that moment, I realized that she had lost trust in both me and in the efficacy of EGFR-TKI therapy.

I recall that her expression softened slightly when I referred her to a dermatologist, advising that, with appropriate skin care, she could still wear makeup. Given the dermatologist’s extensive experience, she may have placed greater trust in them than in me. This incident highlighted my inexperience. I now reflect that, had I communicated the potential adverse effects of afatinib and engaged in collaborative decision-making, I might have preserved her trust.

With the dermatologist’s assistance and the effectiveness of afatinib, Ms. N gradually regained confidence in both me and the treatment. However, 23 months after commencing afatinib, liver metastases were detected. Over the course of 8 years, she underwent several guideline-based therapies, including platinum doublet chemotherapy with bevacizumab, osimertinib, and docetaxel with ramucirumab, ultimately receiving oral 5-fluorouracil-based S-1, the last recommended option in Japanese guideline.

Under Japan’s universal health insurance system, treatment can continue beyond the last recommended option if patients desire. Prior to this, I had frequently discussed at hospital conferences the potential to extend chemotherapy. Simultaneously, over the course of her illness, the Japanese government and the Japan Lung Cancer Society emphasized the significance of shared decision-making. After several years as a respiratory physician, I had the opportunity to participate in a session on shared decision-making at a congress, recognizing a shift in the physician-patient relationship in Japan. As I gained ­experience, I began to cultivate the courage to engage in candid conversations with patients and involve them in decision-making.

“*Your remaining time is limited*.”

When oral S-1 became less effective, I was finally able to communicate this truth: “*There are additional options for chemotherapy, but they would necessitate extended hospital stays. You can decide whether you wish to pursue them*.”

The final treatment decision was reached following several outpatient visits after our discussion. During this time, she engaged in numerous conversations with her family and with me. I made it a point to repeatedly explain her options, aiming to make the decision collaboratively while respecting her wishes.

In the end, we—she and I—chose to prioritize her time with family over hospitalization. Ultimately, as it became increasingly difficult for her to visit our facility, she decided to spend her final days at home.

After I arranged for her to be seen by a home care physician, I visited her only once. Although she appeared weary, our conversation seemed to restore some of her vitality. Despite the facial swelling caused by steroid use, she smiled and told me,

“*I spend every day with my sister and our dog. It’s like a party every day*.”

That was the last time I saw her.

Only 3 weeks after her passing, Japan approved the combination treatment of amivantamab plus lazertinib, as well as amivantamab plus chemotherapy for cases of EGFR-TKI ­resistance.^2^^,^^3^ Had these options been available earlier, they might have been suitable for her. However, we will never know if she would have chosen such a regimen, which carries a 72% incidence of Grade ≥ 3 adverse events.^3^

Currently, first-line EGFR treatment options include three regimens: osimertinib, osimertinib plus chemotherapy, and amivantamab plus lazertinib. As was the case during my residency, we must make treatment decisions for EGFR-mutated lung cancer outside of established guidelines. These advancements in EGFR-TKIs not only prolong survival but also broaden the spectrum of meaningful choices, allowing shared decision-making to evolve from a mere discussion of treatment feasibility to aligning treatment with patients’ values and life goals. Given that studies indicate differing perspectives on treatment between patients and physicians,^4^^,^^5^ fostering dialogue and shared decision-making will be increasingly crucial.

In Japan, we are transitioning from “*Mund Therapie*” to “*Informed Consent*” and “*Shared Decision-Making*.” We are now entering an era where shared decision-making is becoming increasingly vital in selecting treatments for EGFR-mutated lung cancer. Patients will always rely on their physician’s best recommendations. While shared decision-making has its limitations, our responsibility is to propose the most appropriate treatment while honoring each patient’s values and choices.

The past 8 years have witnessed remarkable changes in the physician-patient relationship and a parallel evolution in the treatment of EGFR-mutated lung cancer—with the life of one patient serving as a poignant focal point in this transformation.
